# MR Imaging of the Pelvic Bones: The Current and Cutting-Edge Techniques

**DOI:** 10.5334/jbsr.2874

**Published:** 2022-11-22

**Authors:** Lieve Morbée, Elke Vereecke, Frederiek Laloo, Min Chen, Nele Herregods, Lennart Jans

**Affiliations:** 1 University Hospital Ghent, BE

**Keywords:** Pelvic bones, Magnetic Resonance Imaging (MRI), Gradient echo MRI, Susceptibility weighted MRI, Ultrashort echo time MRI, Synthetic Computed Tomography

## Abstract

This review presents an overview of the spectrum of the current and cutting-edge MRI techniques for pelvic bone imaging in clinical practice. The current MRI sequences and their advantages, disadvantages and usefullness in the imaging of this complex anatomical region are addressed. Finally, cutting-edge techniques are discussed, including susceptibility weighted MRI, ultrashort echo time MRI, zero echo time MRI and a deep learning-based multiparametric MRI technique named ‘synthetic CT,’ creating CT-like images without ionizing radiaton.

**Main Points:**

- GRE, SWI, UTE, ZTE MRI and synthetic CT sequences depict the cortical outline of the bones better in comparison to conventional MR images.
- MRI-based synthetic CT can create HU maps and allows for automated segmentation of pelvic bones.
- The current and cutting-edge MR techniques for bone imaging are complementary in the characterization of a variety of musculoskeletal disorders.

## Introduction

In the imaging assessment of pelvic bones, radiography is the modality of choice for screening purposes, while CT is currently predominantly used for evaluation of bone lesions in areas with complex anatomy such as the pelvis. Soft tissue assessment benefits from an MRI examination as it displays contrast among different tissues. Major strengths of musculoskeletal MRI are its lack of radiation exposure, selective tissue contrast weighting and high contrast resolution, which allow for excellent visualization of bone marrow abnormalities, tearing and degeneration of ligaments and tendons, fibrous and articular cartilage abnormalities, and characterization of fluid collections. On the other hand, direct bone imaging on MRI remains challenging due to the low proton content in bones as well as to very short transverse relaxation times (T2). Cortical fractures may be hard to appreciate on conventional MR images, as well as mineral deposit forming conditions, ossifying conditions, ankylosing and erosive arthropathies with joint erosions or syndesmophytes. Recently several novel techniques emerged to create CT-like images of pelvic bones, such as susceptibility weighted imaging, ultrashort echo time MRI and synthetic CT, including automated segmentation of pelvic bones.

The aim of this review is to present an overview of the current available MRI techniques for pelvic bone imaging in clinical practice.

## Overview of MRI Sequences

### T1-weighted sequence

The T1-weighted (T1W) sequences provide excellent resolution and represent the most ‘anatomical’ of images. T1 relaxation represents the rate at which protons release their absorbed energy – after excitation by the radiofrequency pulse into the transverse plane – into the surrounding tissues and retrieve longitudinal magnetization. The tissue type is one of the most important features determining T1 relaxation times in normal tissues at clinically relevant field strengths [1]. In a simplified way, T1W images most closely approximate the macroscopic appearance of tissues. It is an important sequence for differentiating between normal and abnormal bone marrow. Abnormal bone marrow is iso- or hypo-intense to skeletal muscle. The relative signal intensity of the imaging pixel is determined by the presence or absence of marrow fat in it. Lower T1 signal intensity is expected in cases in which normal marrow is replaced by tumor (marrow replacement) than conditions in which a significant component of marrow fat remains (marrow infiltration) [2]. T1W images are also important for assessment of fracture lines, which stand out from the surrounding bright, usually fatty marrow signal as a low-signal, linear signal abnormality [1]. For the sacroiliac joints the T1W sequence is most commonly used for detecting erosions in spondyloarthritis. At least one T1W sequence without fat suppression should always be performed for correct characterization of bone lesions. Limitations of the routine T1W sequence include the little contrast between cortical bone and joint space, as well as the indistinct margins between cortical lesions and subcortical bone marrow [3].

### Fat suppressed T2 weighted sequence

T2 relaxation time is determined by the mobility of the water protons. More water content in the tissues, increased extracellular fluid, or more random tissue ultrastructure (e.g. trabeculae disrupted by fracture) may contribute to longer T2 relaxation times [1]. Often the radiologist is asked to seek for the presence of edema in bone marrow or soft tissue. If the soft tissue contains fat, this may hamper differentiation of fat and fluid on conventional nonfatsuppressed T2 weighted (T2W) sequence. By applying T2W images with fat suppression high sensitivity can be achieved in diagnosing periosteal edema, bone marrow edema or inflammation related to trauma, infection, tumor infiltration and other bone marrow lesions [4]. However, bone marrow edema on fluid-sensitive sequences is often nonspecific and has a variable etiology. It is difficult to distinguish tumor from surrounding reactive bone marrow edema. Also, for clear anatomy depiction and segmentation this type of imaging faces some problems. The images can be dark, can have intensity inhomogeneity and show similar intensity in different neighboring tissues [5]. Fat suppression can be obtained in a number of different ways: 1) The fat saturation (fat-sat) technique uses the difference in resonance frequency between fat- and water-bound protons by means of frequency selective pulses and destroys the transversal magnetization of fat-bound protons by spoiler gradients. 2) The Short TI Inversion Recovery (STIR) technique is based on the different relaxation behavior of water and fat tissue and uses an inversion pulse to invert the spins of all tissue and fat protons prior to the excitation pulse. 3) The Dixon method is based on the chemical shift (the difference in resonance frequencies between fat- and water-bound protons) and acquires two images in which the signal from fat- and water-protons are ‘in-phase’ and ‘opposed phase’. 4) Hybrid techniques combine several of these fat suppression techniques such as SPIR (spectral presaturation with inversion recovery). Selection of a fat suppression technique depends on the purpose of the fat suppression (contrast enhancement vs tissue characterization) and the quantity of fat in the tissue being examined. Fat saturation is very useful for suppression of signal from a large proportion of fat and for valid acquisition of contrast-enhanced images. The main pitfalls of this technique are sensitivity to magnetic field inhomogeneity and misregistration artifacts. STIR has the benefit of more homogeneous fat suppression, but suffers from relatively decreased signal to noise ratio (SNR) compared to T2W images and the lack of specificity for fat. Opposed-phase imaging is suited for identification of lesions that contain small amounts of fat, but can be unreliable in the detection of small tumors embedded in fatty tissue [4].

### Proton density weighted sequence

**Figure 1:**
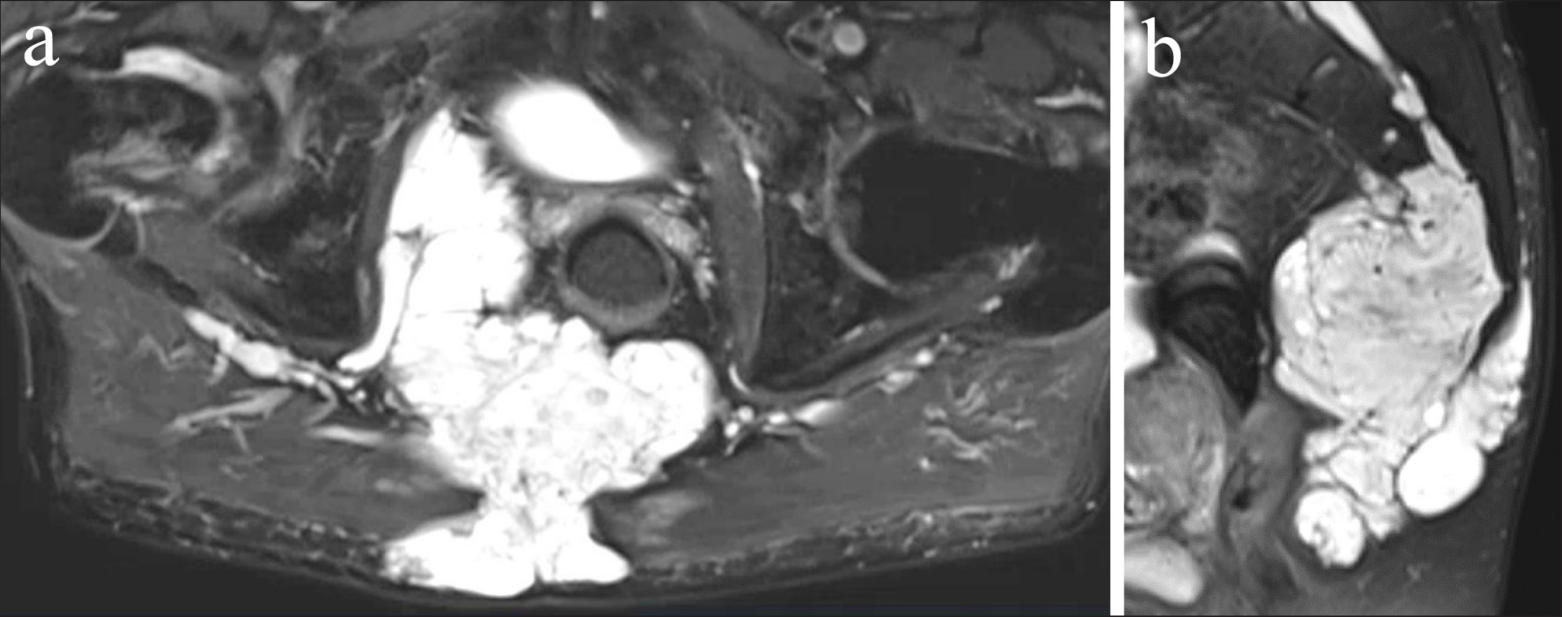
**Fat saturated proton density weighted images.** Axial **(a)** and sagittal **(b)** fat saturated image of a large, lobulated mass in the sacrum and surrounding muscles and fat, compatible with a chordoma in a 82-year-old male. It contains fluid and gelatinous mucoid substance, showing high signal intensity on proton density weighted images.

The proton density weighted sequence is an intermediate sequence that reflects the actual density of protons, as it is correlated with the amount of hydrogen nuclei in the area being imaged, in contrast to the magnetic properties of the hydrogen nuclei. Proton density weighted images are created when the contribution of both T1 and T2 contrast is reduced. They show a long repetition time to eliminate T1 differences because all tissues show complete longitudinal relaxation prior to the next 90 degrees radiofrequency pulse. They have a short echo time to eliminate T2 differences. High proton density tissues appear bright [6]. Proton density weighted images are frequently used to assess pathologies of the joints [6]. They provide detailed anatomy in which the ligaments, labrum and cartilage can be simultaneously assessed with a reasonable scanning time. Structures are displayed in different intensities ranging from high to low: fluid and fatty tissue (i.e. subutaneous fat and trabecular bone), cartilage, muscle, ligament and virtually no signal from the cortical bone. In fat suppressed proton density weighted imaging (Figure 1) the high signal of fluid stands out because the signal from fat is suppressed. Fat suppressed proton density-weighted imaging better depicts marrow abnormalities and soft tissue edema than unsaturated images with similar parameters [7].Limitations of proton density weighted images include an inhomogeneous pattern (due to intrinsic proton density variations), low SNR and inability to clearly define bony borders [6].

### Contrast enhanced sequence

MRI contrast agents have become an indispensable part of contemporary magnetic resonance imaging, as it has been found that the addition of contrast agents in many cases improves sensitivity and/or specificity. In bone imaging intravenous contrast is typically reserved for complicated cases, characterization of bone tumors or in cases in which infection is suspected [4]. Normal bone marrow in adults does not enhance visibly, whereas there may be a significant contrast enhancement in normal marrow of a neonate or a small child. Enhancement in adults occurs only in pathological marrow, which can be accentuated on post-contrast images with fat saturation. Intravenous contrast can better differentiate bone marrow lesions, associated soft tissue or synovial lesions and fluid collections.

### Gradient Echo sequence

**Figure 2:**
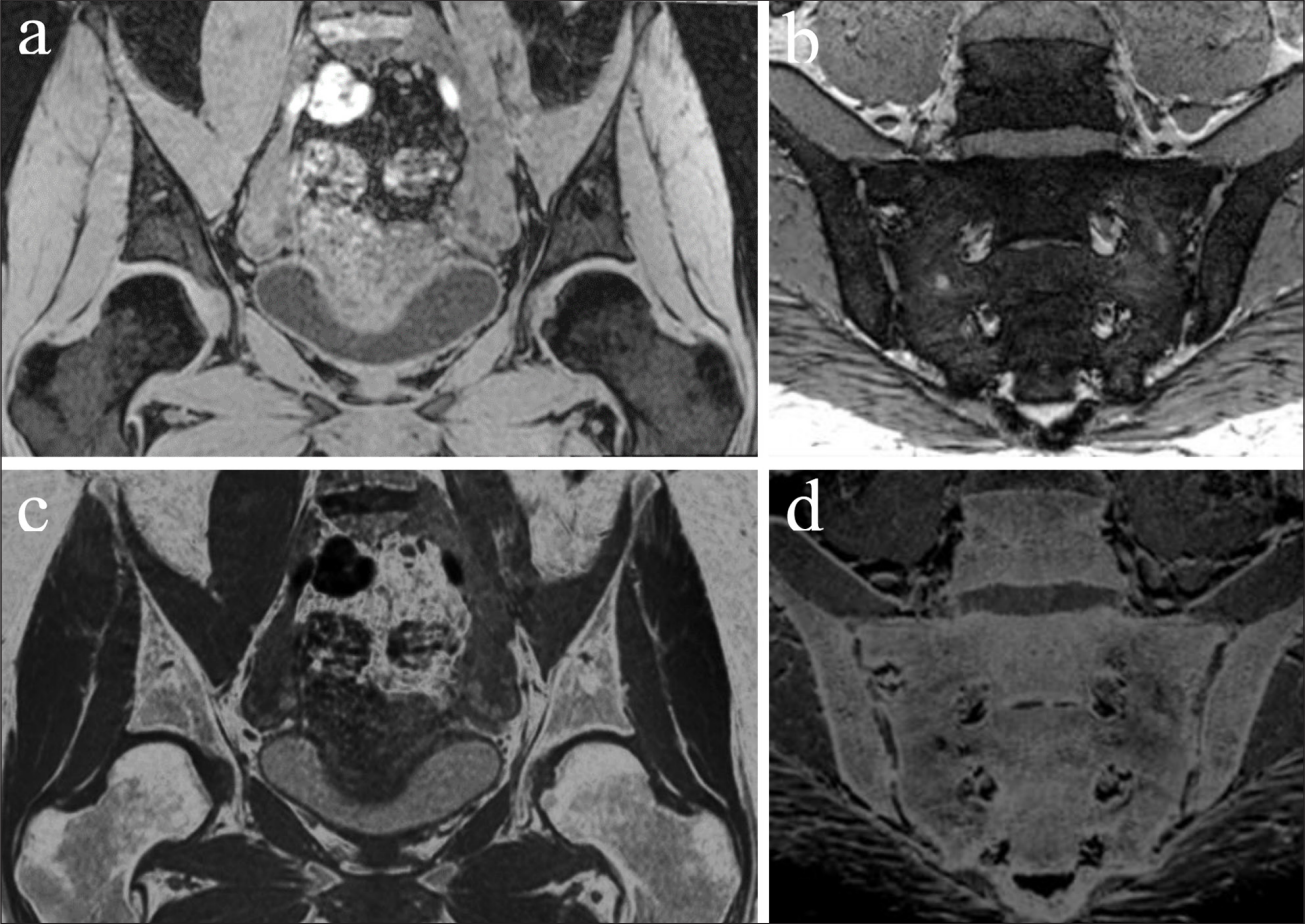
**Gradient echo images and susceptibility weighted images (SWI) (a, b) and with inverted grey-scale (c, d). (a)** Coronal 3D T1-weighted (T1W) spoiled gradient echo image of the femoroacetabular joints. **(b)** Semi-coronal SWI magnitude image of the sacroiliac joints. **(c)** Inverted grey-scale applied on image (a) creating a ‘CT-like’ image. **(d)** Inverted grey-scale applied on image (b) creating a ‘CT-like’ image.

T2* decay underlies all gradient echo (GRE) imaging and helps distinguishing inherently inhomogeneous tissues with magnetic susceptibility effects such as calcium and blood [1]. T2* images are very useful for characterization of pigmented villonodular synovitis (PVNS) and tenosynovial giant cell tumors (TGCT) by the detection of hemosiderin deposition. 3D GRE sequences typically utilize short echo times and relaxation times with optimized radiofrequency flip angles for enhanced bone-soft tissue contrast. They allow the bony margins to stand out because of darkening related to susceptibility of trabeculae [6]. Cortical bone contrast can be indirectly generated from the relief of low-signal bone contours opposed to the surrounding high soft tissue signal by employing grey-scale inversion to provide CT-like images (Figure 2). As such, T1W 3D GRE imaging for bone visualisation remains a qualitative technique. This results in high contrast between joint cavity, cartilage and cortical bone [8]. GRE sequences allow good visualisation of articular cartilage defects and are useful for assessing focal lesions with chondroid matrix [9,10]. Furthermore, 3D sequences have a high spatial resolution, lower partial volume effects, allow for multiplanar reconstruction and have a general, widespread availability in hospitals. In the last few years 3D GRE sequences have been shown to enhance the contrast between soft tissue and bone in different studies of the hips and to facilitate the depiction of erosions of the sacroiliac joints [8,11,12,13]. Among the 3D MRI sequences, the 3D VIBE sequence was most studied in recent years [11,13,14]. It allows for short acquisition times without reducing image quality and generates T1W images with fat suppression [14]. Limitations of 3D GRE sequences include the subjection to artifacts such as intravoxel dephasing and susceptibility to paramagnetic effects [15].

### Susceptibility Weighted sequence

**Figure 3:**
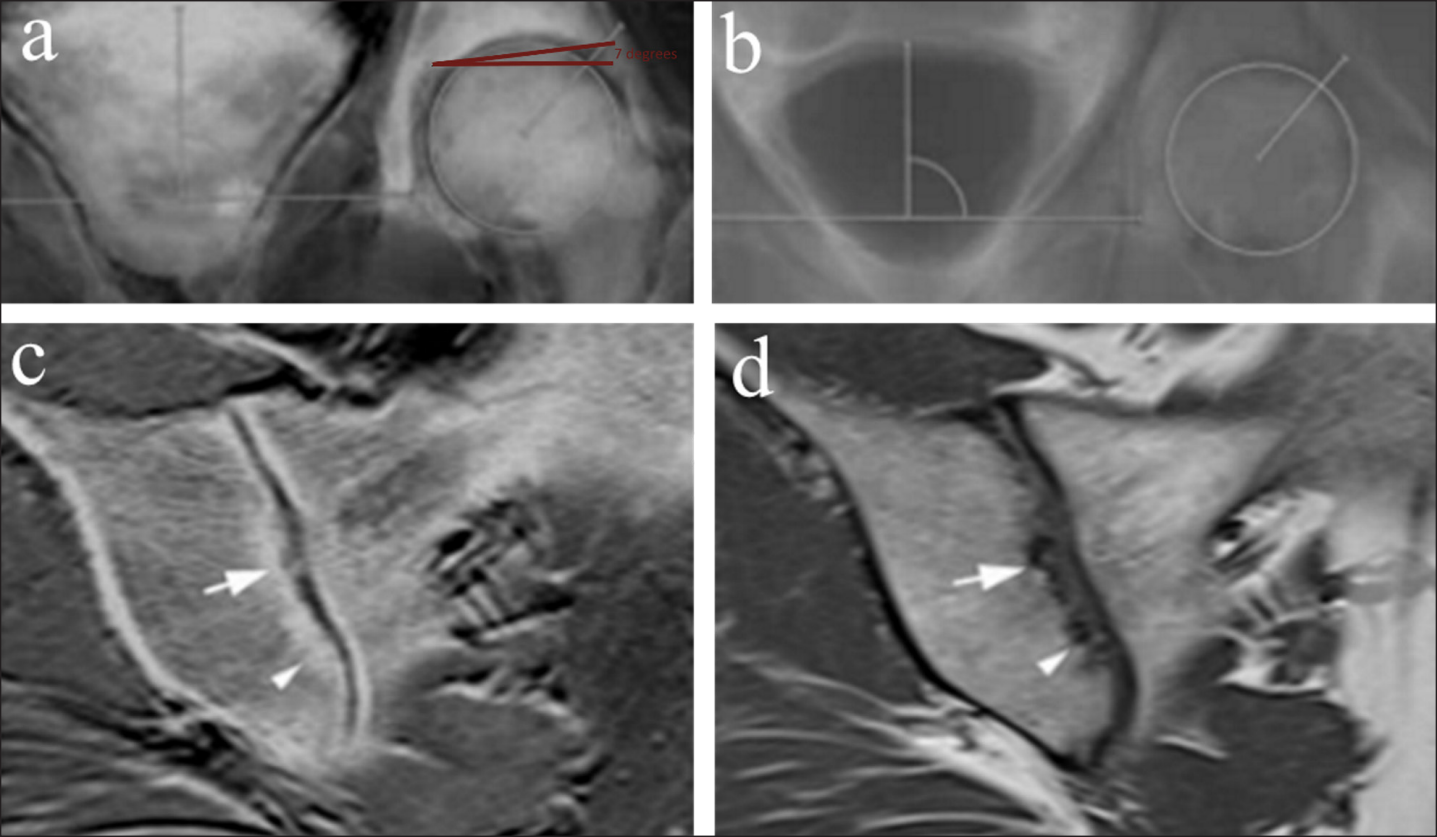
**Susceptibility weighted images (SWI) versus T1-weighted (T1W) images. (a, b)** Coronal maximum intensity projections of SWI MRI magnitude images **(a)** and T1W MRI **(b)** with magnifications and angle measurements. The acetabular index or Tönnis angle (red lines), measurement of acetabular inclination, is an angle assessed by a line drawn from the medial edge of the acetabular sourcil through to the most lateral aspect of the sourcil and by a second line drawn in the horizontal plane of the pelvis (normal range 3°–13°, in this case 7°). This angle could not be measured on T1 because the acetabular sourcil could not be identified reliably. Figure reproduced from Böker et al. [21] under the Creative Commons Attribution 4.0 International License (CC Creative Commons — Attribution 4.0 International — CC BY-NC 4.0, https://creativecommons.org/licenses/by-nc/4.0/). Minor modifications were performed for presentation purposes. **(c, d)** False positive detection of erosion (arrow) in T1 in the sacroiliac joint of a 51-year-old woman with spondylarthrosis. SWI **(c)** shows smooth joint surfaces with mild sclerosis mimicking erosive changes (arrowhead) in T1 **(d)**. Figure reproduced from Deppe et al. [20] under the Creative Commons Attribution 4.0 International License (CC Creative Commons — Attribution 4.0 International — CC BY-NC 4.0, https://creativecommons.org/licenses/by-nc/4.0/). Minor modifications were performed for presentation purposes.

Susceptibility weighted imaging (SWI) (Figures 2 and 3) is a sequence based on the magnetic susceptibility differences between tissues. It is likewise based on GRE sequences, but particularly designed to improve the accuracy of MRI for identifying areas with high susceptibility alterations in the local magnetic field [16]. Reconstructions of magnitude and phase images are feasible. SWI is principally used at the level of the brain to detect micro-haemorrhages, calcifications, iron and deoxyhemoglobin. In contrast to classical GRE sequences, SWI is able to differentiate between deoxygenated blood and calcium [17]. Its use is possible but has not been extended to daily clinical practice because of limitations in spatial resolution and multiple artifacts due to phase-encoding directions, bone-tissue interfaces, flow and increased noise. Also, because of the lack of specific clinical applications and technical questions related to coils design and image analysis the SWI sequence is not routinely applied in the musculoskeletal system [18]. Nevertheless, SWI has been demonstrated to be a feasible approach for characterization of deoxygenated blood and calcium in the musculoskeletal system that can be used as a complementary sequence in the bony pelvis for characterization of complex bone and soft tissue lesions, fracture detection, assessment of calcific tendinopathies and detection of calcifications within bones and joints [19]. Furthermore, it allows reconstructions of CT-like images without radiation exposure. Recently the first study was published using SWI to create CT-like images for detection of structural sacroiliac joint lesions in axial spondyloarthritis, showing SWI depicts erosions and sclerosis more accurately than T1W MRI [20]. Likewise, Böker et al. [21] found that SWI in the hips empowers the reliable assessment of Sharp’s angle, Tönnis angle, lateral center–edge angle of Wiberg and caput-collum-diaphyseal angle in comparison to radiographs with a higher accuracy than conventional MR images. This study demonstrates the utility of an additional SWI sequence in patients with suspected abnormalities of hip morphology, as it provides extra information, without radiation exposure. Intrinsic disadvantages of SWI are the artifacts arising from undesirable magnetic susceptibility sources. Also, SWI-MRI does not provide quantitative Hounsfield Unit (HU) maps in contrast to CT.

### Ultrashort and zero echo time sequence

**Figure 4:**
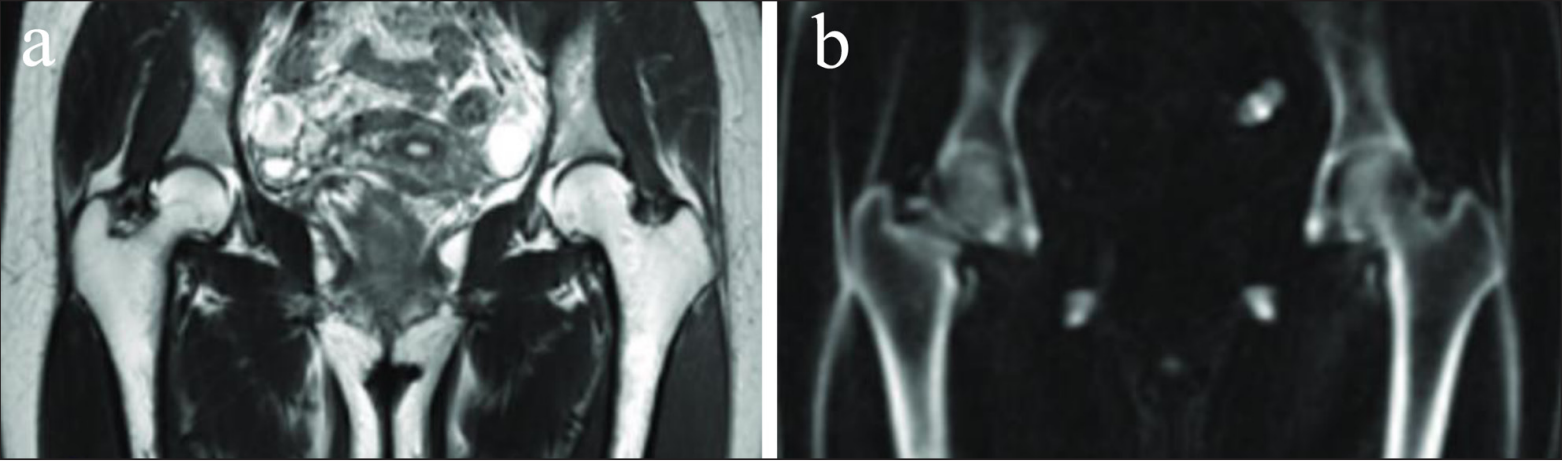
**T2-weighted (T2W) image versus ultrashort echo time (UTE) image.** In vivo imaging of the hip with a 2D T2W fast spin echo sequence **(a)** and 3D Inversion Recovery (IR) UTE Cones sequence **(b)**. Soft tissues are well-suppressed in the 3D IR-UTE Cones image, while they are bright in the T2W images. Figure reproduced from Jerban et al. [23] under the Creative Commons Attribution 4.0 International License (CC Creative Commons — Attribution 4.0 International — CC BY-NC 4.0, https://creativecommons.org/licenses/by-nc/4.0/). Minor modifications were performed for presentation purposes.

**Figure 5:**
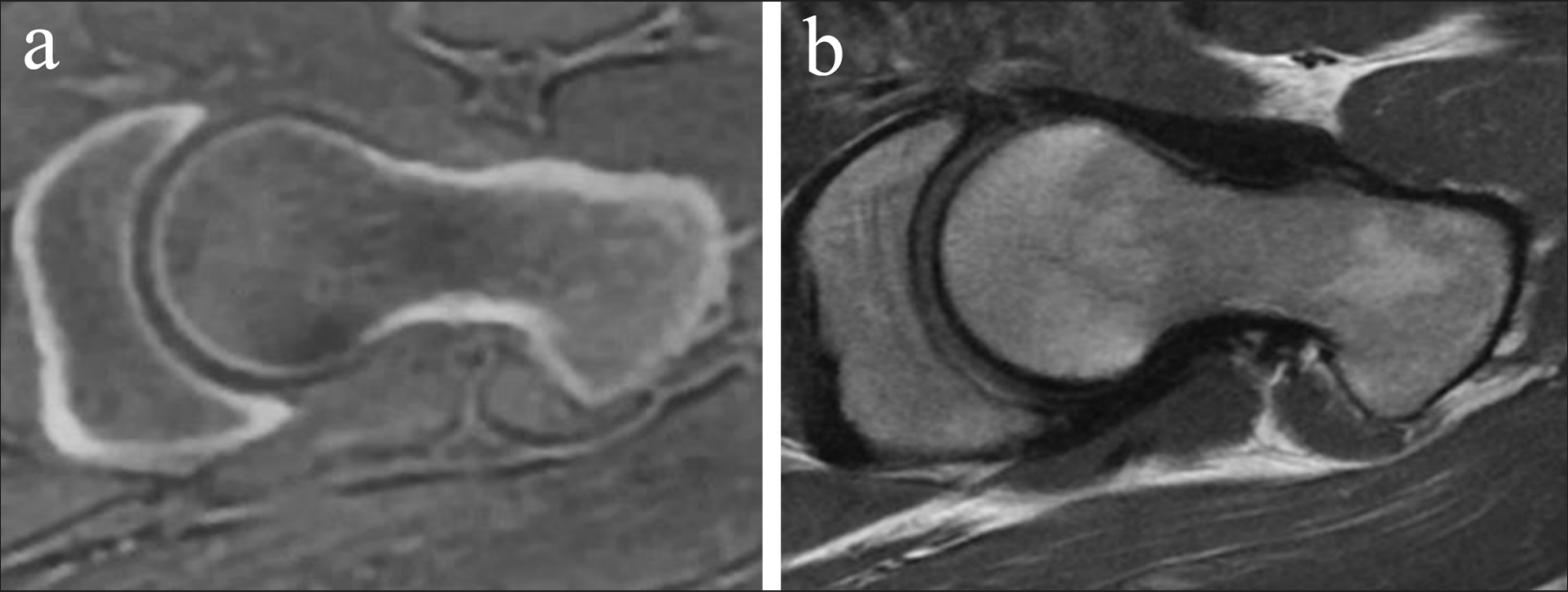
**Zero echo time (ZTE) versus proton density weighted images.** Swiss axial views of the femur are shown in ZTE MRI **(a)** and proton density weighted MRI **(b)**. The hypointensity at the cortical margins renders the cortices difficult to distinguish from the joint capsule or the chondrolabral junction. Courtesy of Ryan Breighner, Weill Cornell Medical College Of Cornell University, New York [25].

In the last decade specialized MR techniques such as ultrashort echo time (UTE) (Figure 4) and zero echo time (ZTE) (Figure 5) MRI have emerged for imaging of cortical bone. They employ dedicated techniques that rapidly capture the sparse signal from short T2 tissues before signal decay. Echo times less than 1ms are realized by rapidly altering the radiofrequency (RF) excitation pulses between transmit to receive modes [22]. UTE allows direct visualization of tissues with very short T2 times such as cortical bone, because it identifies signal from both bound water and pore water in bone (whereas conventional MRI cannot image bound water because the transverse relaxation time is too short), yet with low contrast because of much higher signal from neighboring soft tissues including muscle and bone marrow fat [23]. A variety of methods has been reported to enhance UTE cortical bone contrast by minimizing signal from long T2 tissue components and enabling selective bound water visualization with CT-like bone contrast. These methods include simple image subtraction between UTE and GRE images acquired with a longer TE, IDEAL (Iterative Decomposition Water and Fat with Echo Asymmetry and Least-Squares Estimation) fat suppression and inversion recovery preparation pulses [24]. ZTE MRI also has potential for displaying cortical bone with tissue contrast resembling CT by taking advantage of the very short relaxation time of bone. ZTE is different from UTE by using the readout gradient before the radiofrequency excitation, dismissing the quickly switching gradients between repetition times, with the acquisition of only one echo time dataset [22]. This results in significantly decreased acoustic noise and less artifacts. ZTE and UTE are 3D techniques allowing multiplanar and volumetric reconstructions. They have been utilized for quantitative (e.g. proton density/T2*) assessment of cortical bone and tissues with very short T2 times such as cartilage, tendons, menisci and teeth [24]. UTE and ZTE MRI have been investaged for the evaluation of osseous morphology in different studies of the hip and sacroiliac joint [25,26,27]. However, the use in clinical practice is limited because the technique is prone to false positive bone identification at water-fat interfaces or fascia and requires time-consuming image processing, dedicated scanner hardware and high-end gradients, not available in all hospitals [26,28,29]. Finally, UTE-MRI and ZTE-MRI do not provide quantitative HU maps in contrast to CT.

### MRI-based synthetic CT and automated segmentation

**Figure 6:**
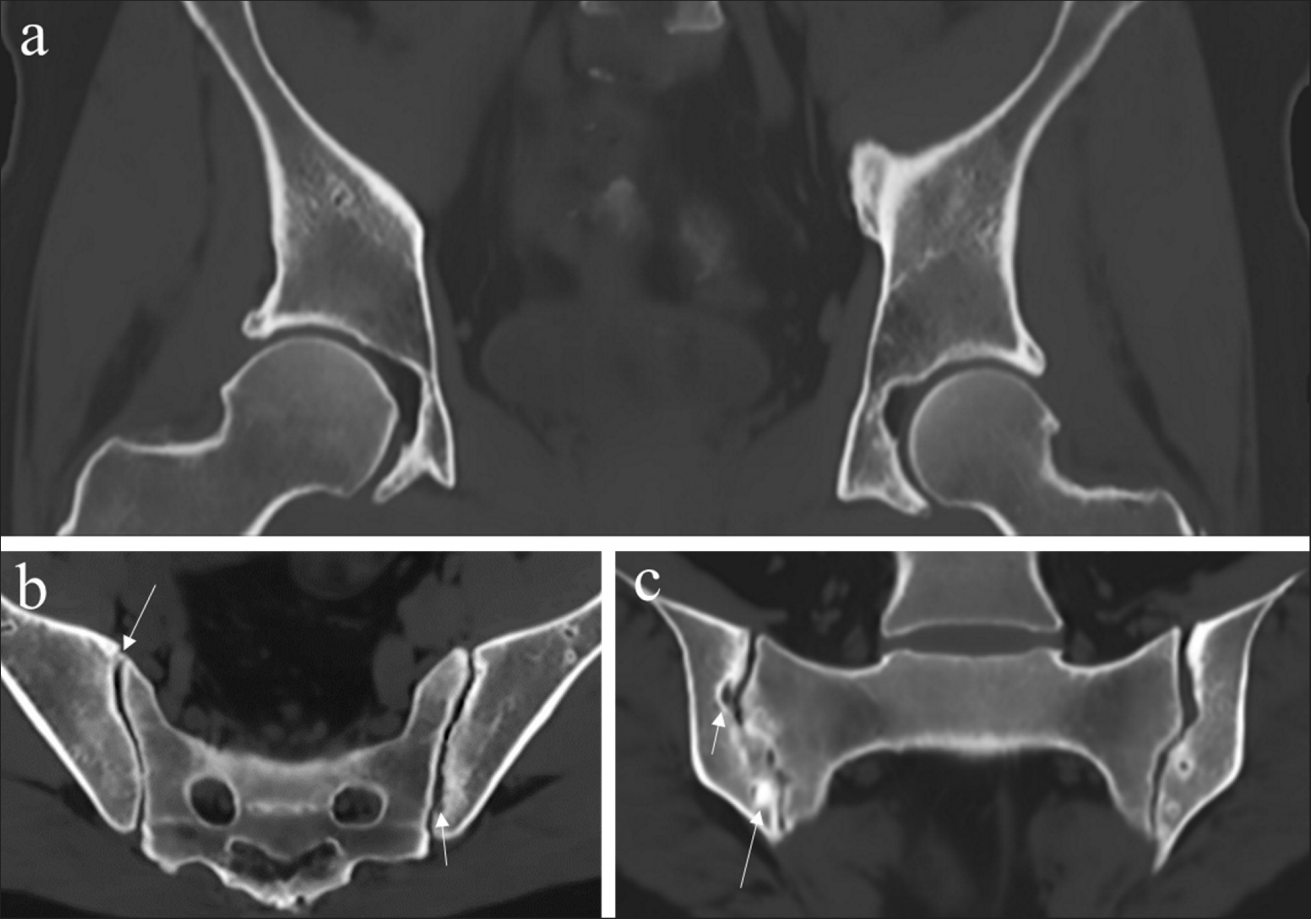
**MRI-based synthetic CT. (a)** Coronal image with bilateral degenerative hip disease with subchondral sclerosis and osteophytes in a 47-year-old male. **(b)** Axial image of erosions (short arrow) with subchondral sclerosis in the left sacroiliac joint and incipient bony bridging in the right sacroiliac joint (long arrow) in a 30-year-old male with sacroiliitis. **(c)** Semicoronal image of the sacroiliac joint shows erosions (short arrow) with subchondral sclerosis (long arrow) in the right sacroiliac joint in a 42-year-old female with sacroiliitis.

**Figure 7:**
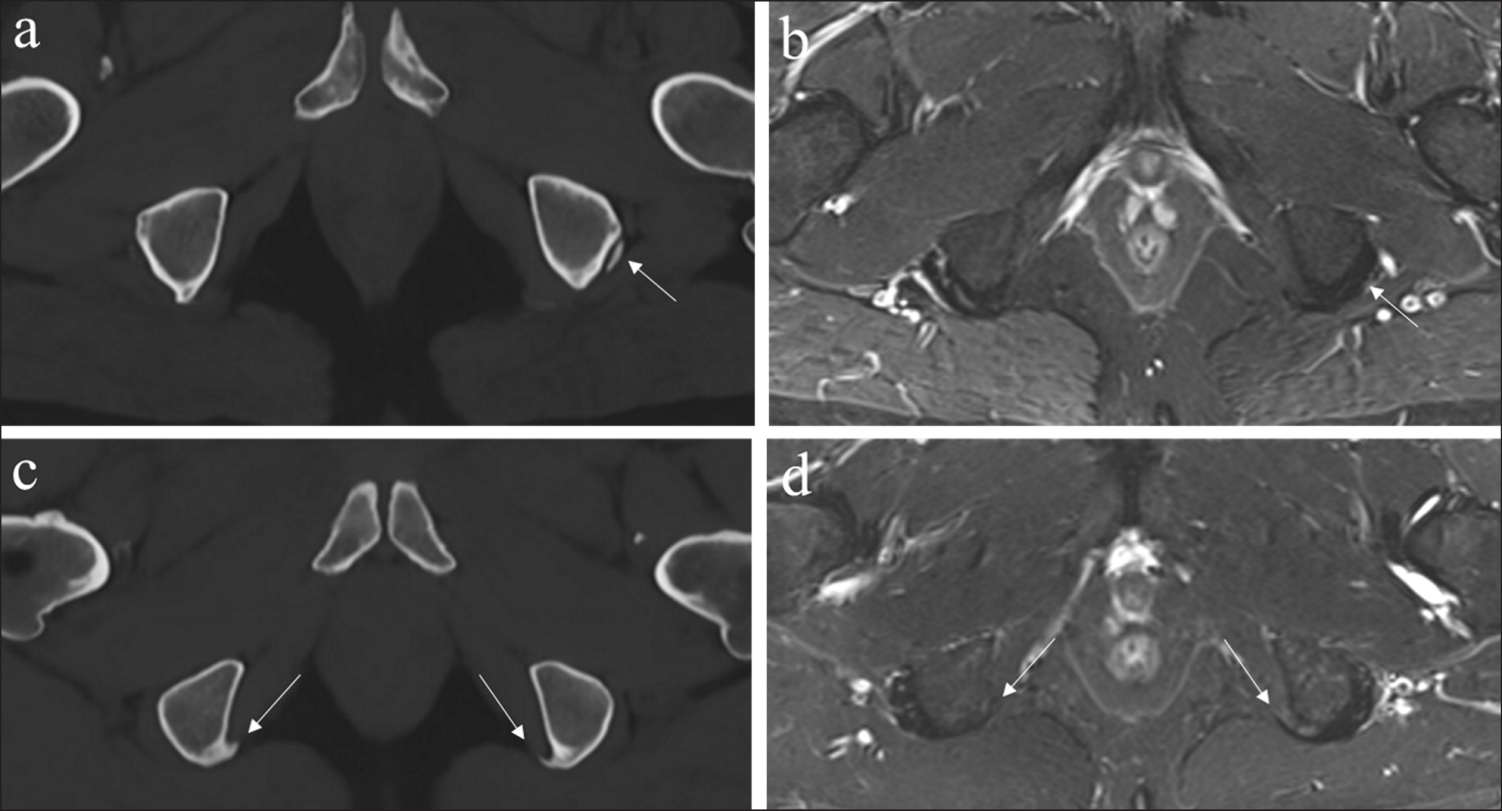
**Incidental findings on sacroiliac joint MRI, depicted on synthetic CT images, but hard to appreciate on the T2 Short TI Inversion Recovery (STIR) images. (a, b)** Old avulsion of the left ischial tuberosity in a 47-year-old male on synthetic CT (a), but hard to depict on the T2 STIR image **(b)**. **(c, d)** Calcifications near the ischial tuberosities, suggestive of calcific enthesopathy, in a 35-yearold male with buttock pain, clearly visible on synthetic CT **(c)**, but less evident on the T2 STIR image **(d)**.

**Figure 8:**
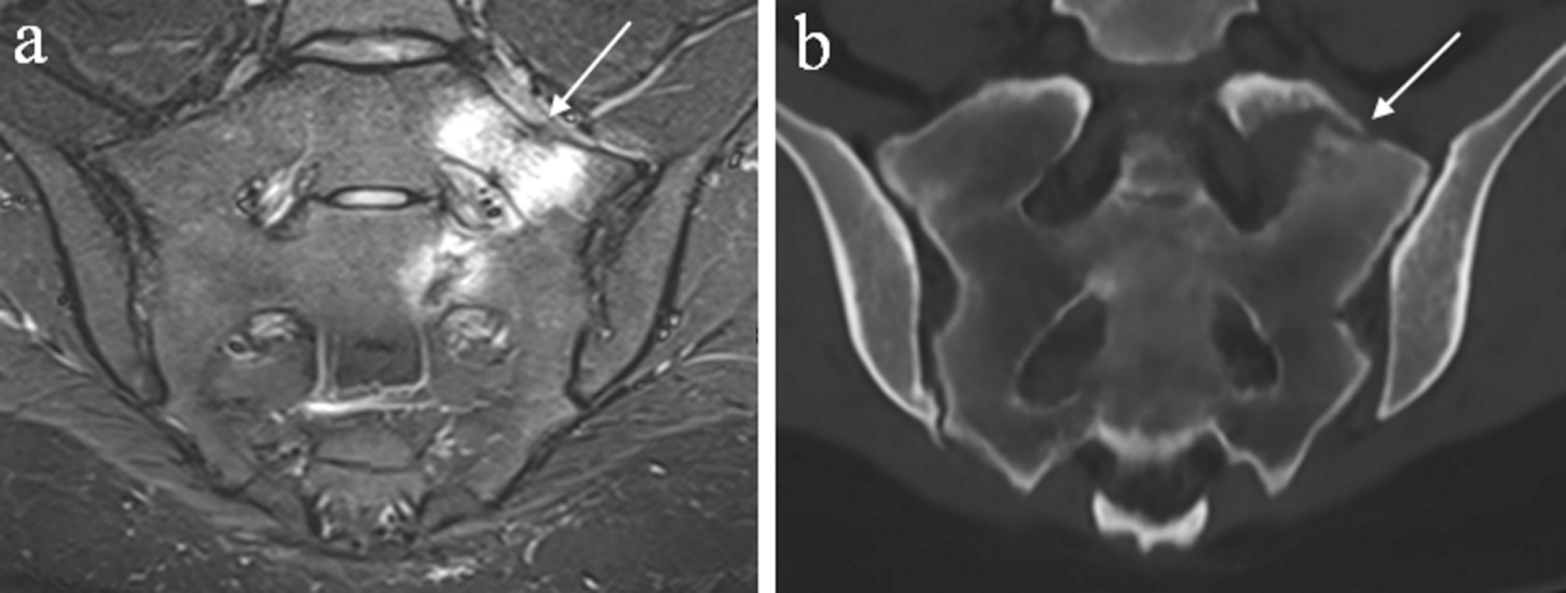
**Synthetic CT and the T2 Short TI Inversion Recovery (STIR) sequence are complementary for diagnosis.** A 22-year-old female with low back pain irradiating towards the left calf shows a left sacral stress fracture with bone marrow edema on the semicoronal STIR image **(a)** and associated cortical disruption on semi-coronal synthetic CT **(b)**.

**Figure 9:**
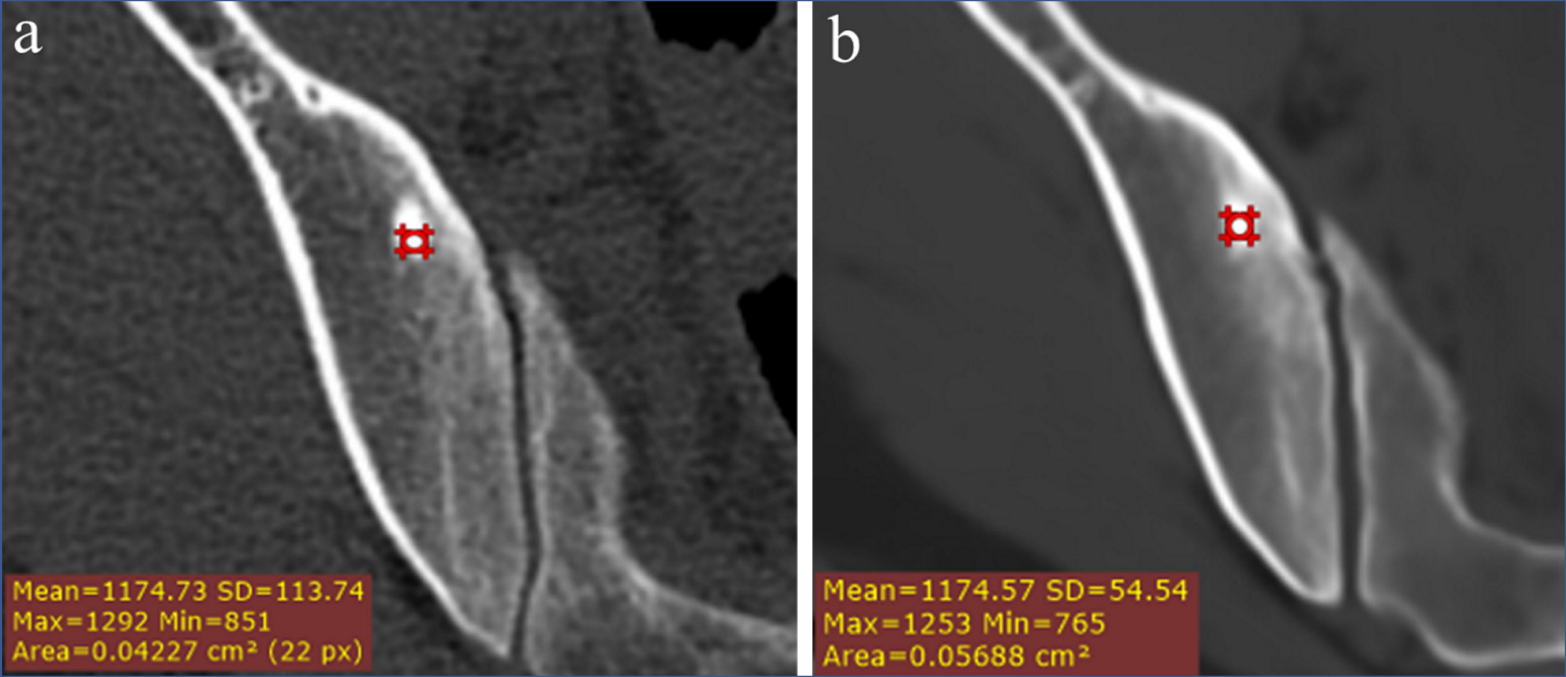
**Hounsfield Units maps.** An incidental enostoma in the right ilium shows the same amount of Hounsfield Units on conventional CT **(a)** as the signal intensities on synthetic CT **(b)**. The measurement of Hounsfield Units on synthetic CT can be very useful, for example in the differentiation of sclerotic lesions. A mean attenuation of 885 HU and a maximum attenuation of 1060 HU are often used as cutoff values in clinical practice – when lower than these reliable thresholds a metastatic lesion is the favored diagnosis [38].

**Figure 10:**
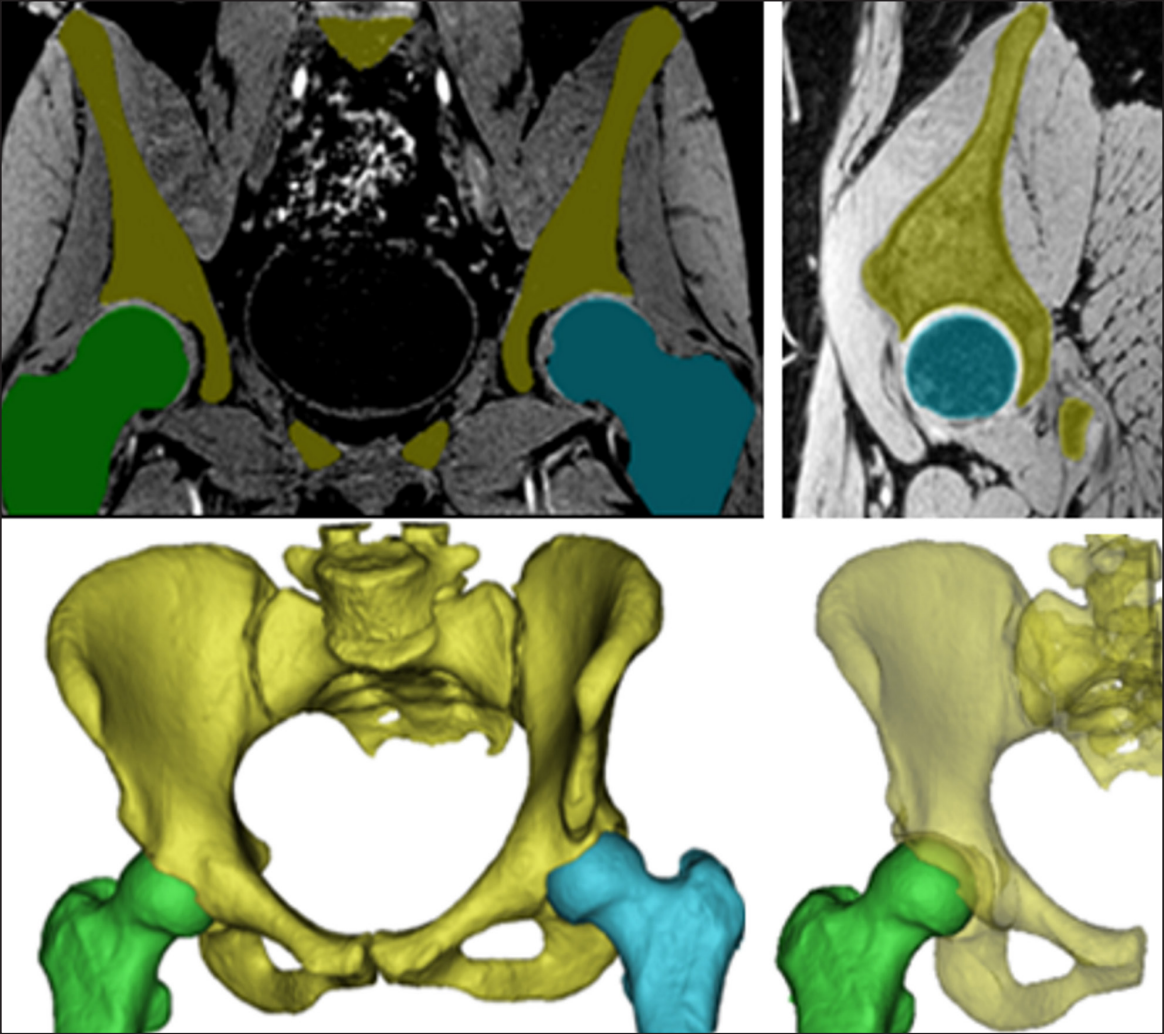
**The 3D nature of the MRI-based synthetic CT reconstruction facilitates multiplanar reconstructions, 3D renderings and automated segmentation** (Courtesy of MRIguidance, University Medical Center Utrecht, The Netherlands; boneMRI v1.4. MRIguidance BV, Utrecht, The Netherlands).

Thanks to the rapid evolution of artificial intelligence a novel technique, ‘synthetic CT’ (or ‘BoneMRI’) (Figure 6, 7, 8, 9, 10) has been developed. This method uses a convolutional neural network, similar to U-Net, with CT scans as the ground truth [30]. The technique needs specialized input data obtained using a generally available sagittal 3D radiofrequency-spoiled T1W multiple gradient sequence, with its parameters accurately chosen with a focus on specific tissue characteristics. Because of the dual-echo images, information about proton density, water and fat fractions, relaxation constants, and susceptibility is intrinsically offered to the deep learning model [30]. As such, radiodensity contrast of osseous structures can be mapped from MRI to CT creating ‘radiograph-like’ and ‘CT-like’ images without ionizing radiation. This technique has the advantage of providing quantitative HU maps like conventional CT and a fully automatic postprocessing process that does not require user input. This technology was clinically validated in the sacroiliac joints, spine and pelvis [30,31,32,33]. In the study of Jans et al. [30], these synthetic CT images in patients with sacroiliitis depicted structural lesions with higher diagnostic accuracy and reliability than T1W MRI, and with reliability comparable to CT. Synthetic CT could also be useful for evaluation and characterization of bone tumors (e.g. nidus in osteoid osteoma, pathological fracture in simple bone cyst, ect.) as has been illustrated previously by Gersing et al. [34]. Furthermore, synthetic CT allows for automated segmentation of the pelvic and femoral bones on the MRI-based synthetic CT images with deep learning software and has the ability for semi-automatic planning of pedicle screw trajectories and screw thicknesses in the lower lumbar spine [35,36]. The ability to visualize the osseous structures in 3D in a similar fashion as traditionally done using CT imaging without radiation and without the need for a separate second examination will be useful in the future, both for diagnostic and therapeutic purposes such as surgical navigation [31,32,37].

## Conclusion

This review discussed the variety of MRI techniques for pelvic bone imaging (summarized in Table 1). While the current MRI sequences each have benefits, pitfalls, and overlapping capabilities in the depiction of pelvic musculoskeletal disorders some cutting-edge MRI techniques for pelvic bones are emerging. These techniques will have a growing role in future years for dedicated bone evaluation with lack of exposure to ionizing radiation, more efficient imaging work-up and improved diagnostic accuracy for a variety of musculoskeletal conditions. Investigations of performance in other body regions constitute interesting future research opportunities.

**Table 1:** Current and cutting-edge MRI techniques for pelvic bone assessment.


SEQUENCE	BENEFITS	PITFALLS

T1	Most ‘anatomical’ image	Limited contrast between cortical bone and joint space

	Fractures, erosions, differentiation bone marrow lesions	Unclear boundaries between cortical lesions and subcortical bone

T2 fs	Bone marrow edema	No clear anatomy

	Inflammation related to trauma, infection, tumor, ect.	No segmentation

PD	Pathology of joints	Inhomogeneous pattern

	Ligaments, labrum, cartilage	Low SNR

		No clear definition of bony borders

Contrast-enhanced	Useful in complicated cases, for characterization of bone tumors and infectious conditions	Transient or allergy-like adverse reactions, nephrogenic systemic fibrosis (rare)

	Higher sensitivity and specificity	Possible gadolinium retention

GRE	Multiplanar reconstructions in 3DGRE	Reliability needs further validation

	High contrast between cartilage and cortical bone; erosion detection	Subject to artifacts

	High spatial resolution and lower partial volume effects than T1 TSE	

	T2* decay useful for focal lesions with chondroid matrix, PVNS, TGCT (blooming artifacts)	

SWI	Direct depiction of osseous structures	Limited experience in the musculoskeletal system

	Grey-scale image inversion -> CT-like images	No radiograph-like images (as synthetic CT)

	No high-end scanner hardware required	Subject to artefacts

	Detection of fractures, erosions, calcifications and complex lesions	

UTE/ZTE	CT-like bone contrast	Limited availability

	Direct depiction of osseous structures	Dedicated postprocessing required

		Lack of specificity for cortical bone

		High end gradients and scanner hardware required

sCT	Radiograph-like, CT-like images and automated segmentation	Limited availability

	Excellent depiction of osseous structures	Requires synthetic CT postprocessing software

	No manual post processing, no high-end scanner hardware required	Vacuum phenomenon can be depicted as bony bridge

	Hounsfield Unit maps	


PD: proton density weighted; T2 fs: fat suppressed T2 weighted; 3D: three-dimensional; GRE: gradient echo; TSE: Turbo spin echo; SWI: susceptibility weighted imaging; UTE: ultrashort echo time; ZTE: zero echo time; sCT: synthetic CT; SNR: signal to noise ratio; PVNS: pigmented villonodular synovitis; TGCT: tenosynovial giant cell tumors.
